# Endocrine Disruptor Bisphenol a Affects the Neurochemical Profile of Nerve Fibers in the Aortic Arch Wall in the Domestic Pig

**DOI:** 10.3390/ijerph19105964

**Published:** 2022-05-13

**Authors:** Liliana Rytel, László Könyves, Slawomir Gonkowski

**Affiliations:** 1 Department of Internal Disease with Clinic, Faculty of Veterinary Medicine, University of Warmia and Mazury, ul. Oczapowskiego 14, 10-719 Olsztyn, Poland; 2 Department of Animal Hygiene, Herd Health and Mobile Clinic, University of Veterinary Medicine, 1078 Budapest, Hungary; konyves.laszlo@univet.hu; 3 Department of Clinical Physiology, Faculty of Veterinary Medicine, University of Warmia and Mazury, 10-719 Olsztyn, Poland; slawekg@uwm.edu.pl

**Keywords:** endocrine disruptors, aorta, nervous system, bisphenol A

## Abstract

Bisphenol A (BPA) is a synthetic compound utilized in industry for the production of various plastics. BPA penetrates into the environment and adversely affects living organisms. Therefore, the influence of various BPA dosages on the neurochemical characteristics of nerve fibers located in the aortic branch wall was investigated in this study utilizing a double immunofluorescence method. It was found that BPA in concentration of 0.5 mg/kg body weight/day causes a clear increase in the density of nerves within aortic branch walls immunoreactive to cocaine- and amphetamine-regulated transcript (CART), calcitonin gene-related peptide (CGRP), neuronal isoform of nitric oxide synthase (nNOS), pituitary adenylate cyclase-activating peptide (PACAP), and vasoactive intestinal polypeptide (VIP). Nerves containing galanin (GAL) and/or somatostatin (SOM) did not change when BPA was introduced into the system. Changes noted after administration of BPA at a dose of 0.05 mg/kg body weight/day were less visible and concerned fibers immunoreactive to CART, CGRP, and/or PACAP. The obtained results show that BPA affects the neurochemical coding of nerves in the aortic branch wall. These fluctuations may be the first signs of the influence of this substance on blood vessels and may also be at the root of the disturbances in the cardiovascular system.

## 1. Introduction

Bisphenol A (BPA) is a synthetic substance belonging to the phenols with two hydroxyphenyl groups [1]. It is widely utilized in the plastic industry and is present in various everyday items (e.g., toys, food containers, furniture, epoxy resins, paints, and dental products, etc.) [2]. Items comprising BPA contacting with drinking water and foodstuffs (such as bottles, food packaging, and cans) are particularly dangerous because BPA has the capacity to penetrate from plastics into its surroundings [3,4]. Due to its common use, BPA is present in the following components: tap water, soil, air, and dust [4]. It may penetrate into human and animal organisms through the digestive tract/system, respiratory system, skin, and placenta (in utero) [5,6,7].

BPA is a strong endocrine disruptor. Because of its similarity to estrogen, BPA binds itself to estrogen receptors and causes multidirectional harmful actions in various organs [8,9]. Previous studies have shown that BPA strongly and adversely impacts on the reproductive, nervous, digestive endocrine, and immune systems [10,11,12]. Moreover, it is known that a high degree of exposure to this substance may be correlated with disturbances in the nervous system development, leading to impairment of higher nervous functions and neurodegenerative diseases, as well as with obesity, diabetes, and neoplastic processes [13,14].

The cardiovascular system is also among the systems that are vulnerable to the influence of BPA. In the light of previous investigations, it is known that BPA influences both the heart and blood vessels. In the heart, BPA enhances oxidative stress reactions in cardiomyocytes [15] and changes nervous structures in the heart wall [16]. These processes probably lead to disturbances in the electrical cardiac rhythm and to the appearance of “triggered activities”, which, in turn, cause BPA-induced heart arrhythmia [17,18]. Previous studies have also described that BPA increases the risk of perimyocarditis and fibrosis [19] in the heart. In turn, within the blood vessels, BPA is an important factor causing the intensification of atherosclerosis processes, which is connected with the expression of BPA-induced adhesion molecules, inflammatory processes, and endoplasmic reticulum stress [20,21]. It is known that BPA causes changes in the functions of the endothelium and the muscular layer in the arteries [22]. It should be highlighted that BPA may not only act on the cardiovascular system directly, but can also cause pathological changes in the heart vessels in the organisms exposed to BPA before birth [23].

The harmful impacts of BPA on the heart and vessels have been confirmed by epidemiological observations in the human population. These observations have reported correlations between the exposure to BPA and the risk of coronary heart disease, myocardial infarction, heart angina, and blood hypertension [24,25]. In people exposed to higher BPA levels. heart attacks, various diseases of the peripheral vessels, and morphological changes in the arterial wall have been noted more frequently [26]. 

However, it should be noted that a number of blood vessel issues directly connected to BPA still remain unclear. Among them, the impact of this substance on nervous structures supplying the vessels seems important and should be further studied. On the other hand, due to the fact that the adverse influence of BPA on the neuronal cells is known from previous studies [12,27], BPA-induced changes in the innervation of vessels may be one of the important mechanisms of the abovementioned disturbances in cardiovascular system functions.

Therefore, the aim of this investigation was to observe the influence of different dosages of BPA on the neurochemical characterization of nerves in the aortic wall. Nerve endings, which are widely distributed throughout the body of humans and animals, secrete a number of biologically active substances. Depending on the tissue in which the ends are located, they can act as neurotransmitters, neuromodulators, and also act as pro- or anti-inflammatory agents. The amount of individual substances in a given tissue may change under the influence of various factors, such as trauma, inflammation, or toxic substances. In response to these factors, a different amount of biologically active substances may be released from the nerve endings, and even substances that are not present in the physiological state may be produced and released. In this study, neurotransmitters that have a proven effect on the cardiovascular system were selected and researched. The fundamental role that CGRP plays in the vascular system is its participation in the cardioprotective mechanism, which is independent of its hypotensive effect. Therefore, it is emphasized that CGRP could protect the vasculature and myocardium against cardiovascular dysfunction [28]. Another described neurotransmitter is nNOS, which is present in the vascular endothelium and importantly contributes to the maintenance of the homeostasis of the cardiovascular system and additionally produces hydrogen peroxide [29]. GAL is described as being able to reduce experimental myocardial injury and to increase cell viability, and, additionally, it inhibits the apoptosis and mitochondrial ROS formation in rat H9c2 cardiomyoblast cells subjected to hypoxia–reoxygenation [30,31]. It is also worth mentioning that VIP and PACAP (both peptides) are involved in the autonomic regulation of the cardiovascular system, where they exert positive inotropic and chronotropic effects and cause coronary vasodilatation. Moreover, PACAP inhibits proliferation of cardiac fibroblasts. Several cardiovascular diseases, such as myocardial fibrosis, heart failure, cardiomyopathy, and pulmonary hypertension, have been found to be associated with changes in the myocardial VIP concentration or with alterations to the affinity, density, and physiological responsiveness of VIP/PACAP receptors [32]. The next mentioned substance by the authors is CART, which is known to be involved in the stress response and has been implicated in the regulation of the cardiovascular system. The direct vasoactive properties of CART in the cerebral circulation and its potential mechanisms of action have been described in [33]. The aim of this publication was to show whether/how the amount of the tested substances changes in the aortic wall under the influence of low and high doses of BPA, which would indicate a clear response of the body to BPA exposure.

It is relatively well established that fluctuations in the chemical profile of nervous structures are the first sign of neurotoxic activities not only caused by BPA but also by other toxic substances [28,29,30,31,32,33,34]. The present study was performed on domestic pigs. Due to the fact that pigs are similar to human organisms in terms of the organization and neurochemical characteristics of the nervous system [29,30,31,32,33,34,35], as well as the biochemical and anatomical properties of the cardiovascular system [30,31,32,33,34,35,36], this species seems to be a very good animal model by which to study the impacts of different chemical factors on the heart and blood vessels [31,32,33,34,35,36,37]. Therefore, this experiment can be treated as the first step towards to a greater understanding of the impact that BPA has on the human cardiovascular system.

## 2. Materials and Methods

This experiment was conducted up on 15 prepubescent female pigs of the Piétrain × Duroc breed bought in the commercial pig fattening farm in the vicinity of Olsztyn (Eden Sp. z.o.o., Gwiżdziny, Poland). The animals were 8 months old at the start of the experiment. During the experiment, the animals were retained in pens appropriate to the age and species of the animal and fed twice a day with commercial complete feed designed for piglets. All the experimental actions were approved by the Ethics Committee in Olsztyn (Poland) responsible for experimental animals (decision numbers 28/2013 of 22 May 2013 and 65/2013/DLZ of 27 November 2013). Before the experiment, the authors performed the following calculations: a power analysis and the significance level, in order to use the smallest number of animals for the research in accordance with the guidelines of the ethical committee so that the obtained results were statistically significant.

The pigs were randomly grouped into three groups of five animals (each group in a separate pen) and subjected to a five-day adaptive period. After this period, the main experimentation commenced. The animals orally received capsules before morning according to the following scheme: (1) control animals received empty capsules; (2) the animals of the bisphenol A I group (BPA I group) received capsules filled with BPA (>99%, catalogue no: 239658-250G, Sigma-Aldrich, Poznan, Poland) at a dosage of 0.05 mg/kg body weight (b.w.)/day; and (3) the animals of the bisphenol A II (BPA II) group received capsules with BPA at a dosage of 0.5 mg/kg b.w./day. The low dose of bisphenol used in the experiment is an acceptable intake dose, while the high dose used in the experiment is ten times this dose (a multiple of this number). The dose was converted per human according to the available dose converters, and these doses were respectively: 0.036 mg/kg and 0.36 mg/kg.

After 28 days, all of the pigs were euthanized. To this aim, the pigs were pre-medicated with Stresnil (Janssen, Beerse, Belgium, 75 μL/kg of b.w., given subcutaneously) and, after about 20 min, treated with an overdose of sodium thiopental (Thiopental, Sandoz, Kundl, Austria, given intravenously).

After euthanasia, the aortic arch was resected from all the pigs. After collection, the samples were fixed in 4% buffered paraformaldehyde (pH 7.4) for 1 h at room temperature (rt), rinsed in phosphate buffer at 5 °C for 3 days, and put into 18% phosphate-buffered sucrose (at 5 °C at least for 3 weeks). Subsequently, fragments of the aortic arches were frozen at −20 °C and cut to sections with a thickness of 10 µm using microtome (HM 525, Microm International, Dreieich, Germany) in cross section. The sections were placed on microscopic slides and retained at −20 °C for further studies.

The neuronal structures in the muscular layer of the aortic arch were studied using double immunofluorescence labeling. Labeling was performed according to the method described previously by Rytel et al. [5]. The method comprised the following stages (performed in rt): (1) drying of the slices (1 h); (2) treatment with solution consisting of 10% normal goat serum, 0.1% bovine serum albumin, 0.01% NaN3, 0.25% Triton x-100, and 0.05% thimerosal in phosphate-buffered saline (PBS) for 1 h; (3) treatment with a combination of two primary antibodies from various species (Table 1): the first was directed against a pan-neuronal marker (protein gene product 9.5 (PGP 9.5)) and the second against one of the other neuronal factors studies, i.e., CART, CGRP, nNOS, PACAP, VIP, GAL, or SOM (overnight); (4) incubation with the combination of secondary antibodies labeled with appropriate fluorochromes (Table 1) to visualize the complex “antigen–primary antibody” (1 h); (5) covering with buffered glycerol. Steps 2–4 were conducted in a humid chamber, and, between the stages of labeling, the tissue fragments were rinsed with PBS (3 times for 10 min). To avoid non-specific labeling, a routine examination of the antibodies’ specificity (including pre-adsorption, omission, and replacement tests) was performed.

The labeled sections of the aortic arches were examined with an Olympus BX51 fluorescence microscope (Olympus, Tokyo, Japan) with the appropriate filter sets. For the evaluation of the density of nerves immunoreactive to the neuronal substances studied, at least 500 nerve fibers that were immunopositive to pan-neuronal marker PGP 9.5 (located in at least 15 sections) were tested for the presence of each of the other substances studied. Nerves immunoreactive to PGP 9.5 were considered as 100%. The glasses were signed with consecutive numbers so that the person counting the fibers did not know which substance was counted. The percentage of nerve fibers immunoreactive to particular substances studied was presented as a mean ± standard error of mean (SEM). To avoid a dual calculation of the same nerve fibers, the sections of aortic wall included in the experiment were separated from each other by at least 400 µm (Table 2) because, according to the literature, the distance between the individual nerve fibers is fairly constant and amounts to 15 µm [38].

The statistical analysis was performed using the one-way analysis of variance (ANOVA) test with Statistica 12 software (StatSoft Inc., Tulsa, OK, USA), and the differences were considered significant at *p* ≤ 0.05. 

## 3. Results

**Figure 1:**
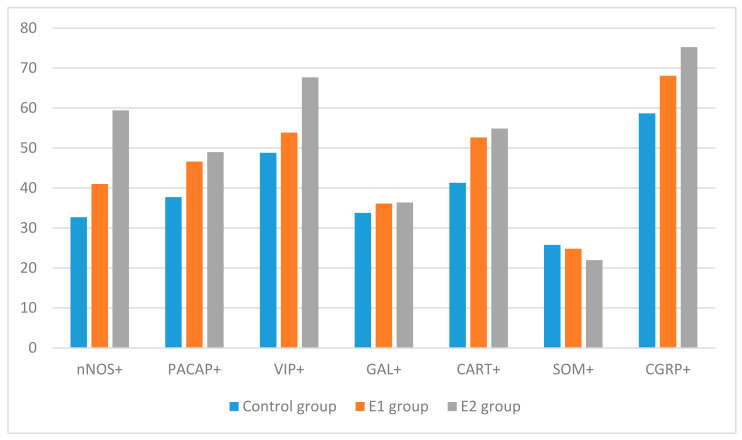
The percentage of nerves containing particular factors in respect of all nerves immunoreactive to pan-neuronal marker PGP 9.5 in control, animals receiving bisphenol A at in low (E1 group) and high dose (E 2 group).

In this investigation, nerves containing all the neuronal factors studied were found both in the animals of the control group and in the animals treated with both dosages of BPA (Figure 1).

Figure 1 The percentage of nerves containing particular factors in respect of all nerves immunoreactive to pan-neuronal marker PGP 9.5 in control animals (control group), in animals receiving bisphenol A at a dose of 0.05 mg/kg body weight/day (BPA I group), and in animals receiving bisphenol A at a dose of 0.5 mg/kg body weight/day (BPA II group). The bar chart represents means and SEM. Statistically significant data (*p* ≤ 0.05) are marked by different letters, and insignificant data are marked by identical letters.

**Figure 2:**
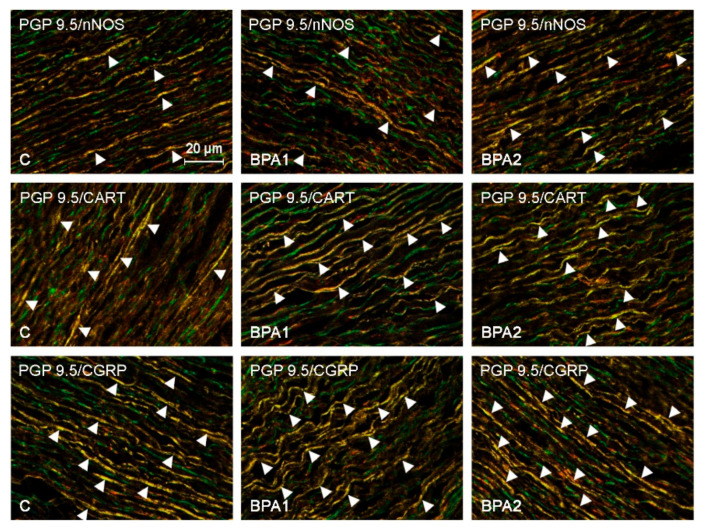
Nerves immunoreactive to PGP 9.5 (stained green) and nitric oxide synthase (nNOS), cocaine- and amphetamine-regulated transcript (CART), and calcitonin gene-related peptide (CGRP) (all stained red) in the in the aortic arch walls of the control animals and the pigs treated with low (BPA1) and high (BPA2) doses of bisphenol A. Nerves simultaneously immunoreactive to PGP 9.5 and nNOS, CART, or CGRP (stained yellow) are indicated with arrowheads.

**Figure 3:**
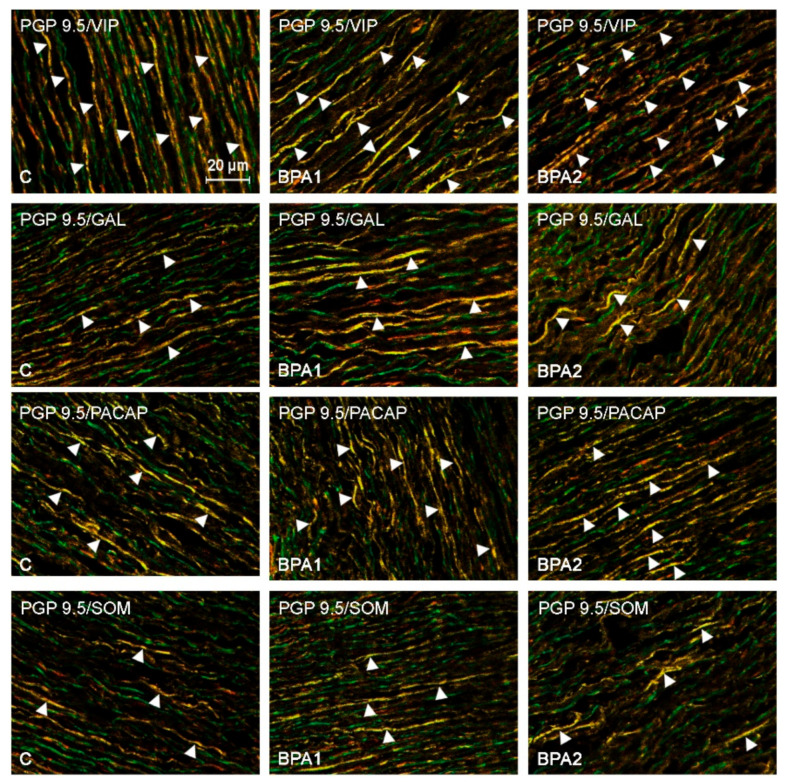
Nerves immunoreactive to PGP 9.5 (stained green) and vasoactive intestinal peptide (VIP), galanin (GAL), pituitary adenylate cyclase-activating peptide (PACAP), and somatostatin (SOM) (all stained red) in the aortic arch walls of the control animals and the pigs treated with low (BPA1) and high (BPA2) doses of bisphenol A. Nerves simultaneously immunoreactive to PGP 9.5 and nNOS, CART, or CGRP (stained yellow) are indicated with arrowheads.

The control animals (Figure 2 and Figure 3) had highest number of nerves immunoreactive to CGRP, which was noted in 58.66 ± 1.99% of all the nerves containing PGP 9.5. A slightly lower percentage of nerves showed immunoreactivity to VIP, CART, and/or PACAP. The percentage of such fibers amounted to 48.79 ± 2.34%, 41.30 ± 1.45%, and 37.70 ± 3.07% of all PGP 9.5-positive nerves, respectively (Figure 1, Figure 2 and Figure 3). An even smaller number of nerve fibers contained GAL (33.76 ± 2.09%) and/or nNOS (32.68 ± 2.31%). The least abundant were the fibers immunoreactive to SOM, which were found in 25.76 ± 0.96% of all the nerves immunoreactive to PGP 9.5 (Figure 1).

The results showed that both of the BPA dosages used in this study affected the number of nerves containing the majority of substances investigated, and these changes clearly depended on the BPA dose and type of neuronal factor (Figure 1 and Figure 2 and Table 2, Table 3 and Table 4). Generally, fluctuations in the number of nerves containing particular substances were more visible after administration of higher dosages of BPA (Figure 1).

The most visible changes were found in the case of nerves containing CART (Figure 1). The number of such fibers increased to 52.65 ± 2.27% of all the PGP 9.5-positive nerves in the animals receiving a lower dosage of BPA, and to 54.86 ± 2.57% in the pigs receiving a higher dosage of BPA. In comparison to the control animals, these values were higher by about 11 percentage points (pp) (in the animals of the BPA I group) and by about 13 pp (in the animals of the BPA II group). Clearly visible BPA-induced fluctuations in the number of fibers also applied to fibers containing CGRP (Figure 1), PACAP (Figure 1), and/or nNOS (Figure 1). The number of CGRP-positive fibers in the animals receiving lower doses of BPA amounted to 68.07 ± 2.46% of all the PGP 9.5-positive nerves (an increase of about 10 pp compared to the control animals), and, in the pigs receiving higher BPA dosages, this value reached 75.24 ± 1.87% (an increase of about 17 pp in comparison to the control animals). The number of PACAP-like immunoreactive (LI) nerves reached 46.60 ± 3.34% (an increase of about 9 pp) in the pigs of the BPA I group, and 48.97 ± 2.25% (an increase of about 11 pp) in the animas of the BPA II group. The changes observed in the case of nNOS-positive fibers noted under the influence of a lower BPA dose were similar. The number of such fibers in the BPA I group reached 40.99 ± 3.22% of all the nerves immunopositive to PGP 9.5, and there were no statistically significantly differences between this value and the value noted in the control animals. However, the higher BPA dosage resulted in visible changes in the number of nerves immunoreactive to nNOS. In the animals of the BPA II group, nNOS was found in 59.42 ± 2.99% of all the PGP 9.5–LI structures (an increase of about 27 pp in comparison to control animals) (Figure 2).

Less visible fluctuations were observed in the case of fibers immunoreactive to VIP (Figure 1). The number of VIP–LI nerves in the BPA I group reached 53.86 ± 2.13% of all the PGP 9.5-positive nerves and was not statistically or significantly different from the number noted in the control animals. In turn, in the animals of the BPA II group, the number of VIP-LI nerves amounted to 67.66 ± 2.99% (an increase of about 9 pp in comparison to the control group). 

During these investigations, the influence of BPA on the number of fibers immunoreactive to SOM (Figure 1) and/or GAL (Figure 1) was not observed (Figure 3). The number of SOM–LI nerves amounted to 24.79 ± 1.89% and 21.97 ± 1.71% of all the PGP 9.5-positive nerves in the animals of the BPA I group and the BPA II group, respectively; the number of GAL-positive nerves amounted to 36.10 ± 2.01% of all the PGP 9.5–LI structures under the impact of lower dosages of BPA, and 36.38 ± 1.71% in the pigs receiving higher dosages of BPA. The number of nerves containing SOM and/or GAL noted after administration of BPA was not significantly statistically different from the values noted in the animals of the control group.

In the present study, BPA-induced changes in the morphological features of nerve fibers were not found. The nerves in the control animals, as well as in those in the animals treated with BPA, were rather thin, but clearly visible.

## 4. Discussion

The present results indicate that the dosages of BPA used in this study caused fluctuations in the neurochemical coding of nerve fibers in the wall of the aortic arch, and the intensification of these changes depended on the dosages of BPA. The experiment was to show whether the acceptable dose would cause changes in the chemical coding of extrinsic aortic innervation as compared to the control group; whether a high dose had such effects; and, if there were changes, to what extent the amount of biologically active substances changed in relation to the control group. According to the authors, it is particularly important to show possible changes in animals receiving a dose acceptable for consumption.

It should be pointed out that many countries have introduced restrictions on the use of BPA due to its harmfulness to living organisms [39]. Such restrictions mainly concern items intended for children (bottles, pacifiers, and toys) because young organisms are particularly sensitive to the toxic effects of BPA [40,41]. The regulations of the majority of countries have introduced the concept of a tolerable daily intake (TDI) dosage or reference dosage of BPA, which should be safe for living organisms [42]. A lower dosage included in this study is considered to be such a dose in some countries thus far [43], although the European Food Safety Authority (EFSA) decreased the TDI from 0.05 mg/kg/day to 4 μg/kg b.w./day because some studies have shown that a dosage of 0.05 mg/kg b.w./day affects the immune system [44]. The results of this experiment have shown that such doses change the neurochemical coding of fibers in the aortic wall to a small extent. On the other hand, the increase in the number of fibers containing CART, CGRP, and/or PACAP under the influence of BPA at a dosage of 0.05 mg/kg b.w./day noted in the present study, as well as in previous observations in which the impact of such a dosage on the nervous structures supplying the internal organs has been found [45], strongly suggest that such a dose of BPA is not completely neutral for use in humans and animals. Therefore, the EFSA decision concerning a decrease in the TDI dosage for BPA was correct. Of course, the dosages of BPA to which living organisms are exposed in everyday life are usually lower than 0.05 mg/kg b.w./day [46], but some situations can foster even higher levels of exposure. For example, previous studies have shown that people with numerous dental fillings of an older type made with materials containing BPA may be exposed to a dose of 30 mg/day [47]. 

It is known that active substances, the contents of which have apparently changed in the nerve structures, are usually involved in processes arising under the impact of BPA on living organisms [39,40,41,42,43,44,45]. Such fluctuations in the number of nerves containing particular substances may result from changes in the synthesis of active factors in neuronal cells and/or their transport from the perikarya to nerve endings [11]. However, due to the fact that this study is the first investigation on BPA’s impact on the aortic wall, the exact reasons for the changes noted in the number of fibers containing particular neuronal substances are not clear. It is worth mentioning that there are very few reports in the literature describing the number and length of nerve fibers, especially in internal organs. Most reports focus on the presence or absence of specific types of fibers rather than quantifying them. To the best of the authors’ knowledge, one independent study found that the distance between the bundles of nerves in large and small arterial vessels was fairly constant, about 15 µm [38].

In the present study, the most visible changes concerned nerves immunoreactive to CART. The roles of this substance in regulatory reactions within the blood vessel wall still remain unclear, especially outside the brain. It is known that a relatively large number of CART–LI neuronal cells are located in the nucleus of the solitary tract and the postrema area, which are known as potent cardiovascular centers [48]. Moreover, the participation of CART in cerebral vasoconstriction has been described [33]. Other experiments have found that supplying this peptide to the central nervous system results in an increase in arterial blood pressure [49]. Therefore, the increase in the number of CART–LI nerves observed in this investigation may be connected with participation of BPA in processes leading to hypertension, which is known from previous studies [50]. Of course, the BPA-induced increase in the number of nerves containing CART can be connected with other mechanisms. Namely, CART is known as a neuroprotective substance that activates antioxidant pathways [51], preserves of mitochondrial function [50], and modulates the immune system [52]. Thus, the observed changes may be connected with relatively well-known pro-inflammatory and neurotoxic properties of BPA [53], and the increase in the number of CART–LI fibers was the response of the organism to BPA’s influence, aiming at the maintenance of homeostasis. It is all the more likely that the neurotoxic and pro-0inflammatory activities of BPA are mainly based on the activation of oxidative stress reactions and disturbances of mitochondrial functions [54,55], i.e., processes opposite to CART activity. 

The next population of nerve fibers, the number of which clearly increased after the treatment with higher dosages of BPA in the present experiment, were fibers containing CGRP. CGRP, similar to CART, is known as a potent protective factor. Previous studies have shown that this substance protects aorta endothelial cells during oxidative injury [56], and also takes part in neuroprotective processes in the central and peripheral nervous systems [57]. It is also known that CGRP takes part in immune reactions and blocks the synthesis of tumor necrosis factor α (TNF-α), an important mediator of inflammatory reactions [58]. Therefore, the changes observed in this study may be connected with the pro-inflammatory actions of BPA. Interestingly, CGRP, contrary to CART, is a potent vasodilator factor. CGRP-induced relaxation has been described not only in the aorta [59], but also in other peripheral blood vessels [60]. The increase in the number of CGRP–LI nerves observed in this study can be connected with the relaxant activity of BPA on the smooth muscles, as is relatively well known from previous investigations [61]. Moreover, changes in the nerves immunoreactive to CGRP may also result from the pro-inflammatory action of BPA [62] because, as is well known, inflammation is accompanied by vasodilation resulting in hyperemia [63]. It may be surprising that the action of BPA caused a simultaneous increase in the number of fibers containing factors causing vasoconstriction (CART) and relaxation (CGRP). The mechanisms of this phenomenon are not clear. However, similar reactions to BPA have been observed in the nervous structures within the gastrointestinal tract and uterus, where administration of BPA resulted in a simultaneous increase in the number of nerves containing substances inducing and inhibiting the muscular activity or pro- and anti-inflammatory factors [10,11]. It should be also noted that CGRP is a key substance taking part in sensory and pain stimuli conduction [64]. However, a connection between the increase in the number of CGRP-LI fibers and this function is unlikely because the BPA dosages used in this investigation were relatively small and did not result in a painful reaction, which was supported by the fact that pigs did not show any clinical signs during investigation. 

The next group of fibers, in which the number clearly changed after administration of BPA at a dosage of 0.5 mg/kg b.w./day, included the fibers containing nNOS, which is a marker of nitrergic nerves [5]. Nitric oxide (NO) in the arteries (similar to CGRP) shows relaxant activity, and, therefore, it is considered to be one of the most important vasodilators [65,66]. Thus, the increase in the number of nerves containing nNOS may result from the influence of BPA on the aortic muscles. However, NO is also a factor that affects the nervous system. Interestingly, previous studies have reported both neuroprotective and neurotoxic properties of NO [67]. On the one hand, NO reduces neuronal degeneration by ameliorating inflammatory processes [68] and influences the expression of the vital factors by neuronal cells [69]. However, on the other hand, it is known that pathological and toxic factors lead to excessive synthesis of NO, which induces oxidative stress processes and therefore causes neuronal cell injury [67]. Moreover, NO participates in the regulation of immune system functions. Previous investigations have noted that NO can be both a pro- and anti-inflammatory substance depending on the kind of inflammation and type of inflamed organ, wherein the cardiovascular system’s anti-inflammatory and anti-proliferative properties predominate [70,71]. Thus, the changes in the number of nNOS–LI nerves may result from the pro-inflammatory and neurotoxic properties of BPA and may be connected with the direct influence of BPA on the nervous system (the toxic properties of NO) or reactions leading to homeostasis maintenance (the neuroprotective activity of NO).

In turn, VIP and PACAP, the levels of which also increased in the aortic wall nerve fibers after administration of BPA, are substances with high homology (about 68%) to each other [72]. Thus, their participation in the regulation of blood vessel activity is similar. VIP and PACAP have been described as important vasodilators and vasodepressors in the cardiovascular system [73,74]. Moreover, they play important roles in protective reactions [75] and participate in the modulation of immune processes under the influence of various pathological factors [76]. The abovementioned activities of PACAP and VIP were probably at the heart of the fluctuations in the number of fibers containing these factors observed in this study.

In the present investigation, BPA did not change the number of fibers containing GAL and/or SOM. This strongly suggests that GAL and SOM, although playing various functions in the regulation of blood vessel activity [77], do not participate in processes triggered in the aorta by exposure to BPA.

## 5. Conclusions

The results show that BPA administered for relatively short period affects the neurochemical coding of nerves in the aortic arch wall. These changes in the innervation are the first signs of the impact of BPA on the aorta and are probably at the heart of BPA-induced disturbances in the cardiovascular system. Due to the fact that this study is the first investigation concerning the impact of BPA on the aortic nerve fibers, the determination of the exact mechanisms causing the observed changes is difficult. Considering the functions of the studied neuronal factors that take part in protective and immunological processes, it can be expected that the changes noted in this study can be connected with the pro-inflammatory and neurotoxic actions of BPA. However, further investigations are essential to confirm such hypothesis.

## Figures and Tables

**Table 1:** Description of the antibodies.

Antigen	Species of Origin	Code	Dilution	Supplier
PRIMARY ANTIBODIES				
PGP 9.5	Mouse	ab72911	1:1000	Abcam
GAL	Rabbit	AB2233	1:2000	Milipore
nNOS	Rabbit	AB5380	1:4000	Chemicon
VIP	Rabbit	VA 1285	1:4000	Biogene
PACAP	Rabbit	ab216627	1:4000	Abcam
CGRP	Rabbit	AB5920	1:4000	AbDserotec
CART	Rabbit	HPA046278	1:2000	Merck
SOM	Rabbit	ab111912	1:2000	Abcam
SECONDARY ANTIBODIES				
Alexa Fluor 546	Donkey Anti-Rabbit	A10040	1:1000	Invitrogen
Alexa Fluor 488	Donkey Anti-Mouse	A21202	1:1000	Invitrogen

**Table 2:** The neurochemical characterization of PGP 9.5-positive fibers in the muscle layer of the aortic arch of the control animals (control group). The results are presented as the percentage of fibers showing the activity of specific substances towards PGP 9.5+.

Control Group							
Number of Animals	PGP 9.5+/ GAL+	PGP 9.5+/ SOM+	PGP 9.5+/ VIP+	PGP 9.5+/ nNOS+	PGP 9.5+/ PACAP+	PGP 9.5+/ CGRP+	PGP 9.5+/ CART+
1	35.03	25.18	45.08	39.05	47.96	64.99	46.17
2	37.38	27.56	52.82	36.73	30.08	54.58	40.07
3	38.56	23.08	52.17	32.17	32.95	54.44	42.96
4	27.82	28.32	52.48	27.19	39.44	60.76	38.75
5	29.99	24.68	41.38	28.24	38.05	58.51	38.54
Minimum	27.82	23.08	41.38	27.19	30.08	54.44	38.54
Maximum	38.56	28.32	52.82	39.05	47.96	64.99	46.17
Mean	33.76	25.76	48.79	32.68	37.70	58.66	41.30
SEM	2.09	0.96	2.34	2.31	3.07	1.99	1.45

**Table 3:** The neurochemical characterization of PGP 9.5-positive fibers in the muscle layer of the aortic arch of pigs treated with a low (E1 group) dose of bisphenol A. The results are presented as the percentage of fibers showing the activity of specific substances towards PGP 9.5+.

E1 Group							
Number of Animals	PGP 9.5+/ GAL+	PGP 9.5+/ SOM+	PGP 9.5+/ VIP+	PGP 9.5+/ nNOS+	PGP 9.5+/ PACAP+	PGP 9.5+/ CGRP+	PGP 9.5+/ CART+
1	36.99	24.25	52.97	44.73	43.48	75.27	47.81
2	29.92	19.87	52.98	42.93	38.09	69.94	59.52
3	41.63	21.67	60.63	29.24	51.25	67.94	51.00
4	38.38	28.50	47.41	39.22	43.17	60.02	48.59
5	33.59	29.66	55.31	48.85	56.99	67.14	56.34
Minimum	29.92	19.87	47.41	29.24	38.09	60.02	47.81
Maximum	41.63	29.66	60.63	48.85	56.99	75.27	59.52
Mean	36.10	24.79	53.86	40.99	46.60	68.07	52.65
SEM	2.01	1.89	2.13	3.32	3.34	2.46	2.27

**Table 4:** The neurochemical characterization of PGP 9.5-positive fibers in the muscle layer of the aortic arch of pigs treated with a high (E2) dose of bisphenol A. The results are presented as the percentage of fibers showing the activity of specific substances towards PGP 9.5+.

E2 Group							
Number of Animals	PGP 9.5+/ GAL+	PGP 9.5+/ SOM+	PGP 9.5+/ VIP+	PGP 9.5+/ nNOS+	PGP 9.5+/ PACAP+	PGP 9.5+/ CGRP+	PGP 9.5+/ CART+
1	39.78	26.50	64.95	65.03	43.30	77.31	54.13
2	29.91	22.32	77.93	58.97	56.51	78.47	62.39
3	37.98	16.17	59.53	67.24	50.76	78.99	57.48
4	36.22	23.84	67.94	51.87	46.17	70.62	46.71
5	38.02	21.04	67.46	54.01	48.10	70.80	53.58
Minimum	29.91	16.17	59.53	51.87	43.30	70.62	46.71
Maximum	39.78	26.50	77.93	67.24	56.51	78.99	62.39
Mean	36.38	21.97	67.66	59.42	48.97	75.24	54.86
SEM	1.71	1.71	2.99	2.99	2.25	1.87	2.57

## Data Availability

The data presented in this study are available on request from the corresponding author.
